# Benefits of a Working Memory Training Program for Inattention in Daily Life: A Systematic Review and Meta-Analysis

**DOI:** 10.1371/journal.pone.0119522

**Published:** 2015-03-20

**Authors:** Megan Spencer-Smith, Torkel Klingberg

**Affiliations:** 1 Department of Neuroscience, Karolinska Institutet, Stockholm, Sweden; 2 School of Psychological Sciences, Monash University, Melbourne, Victoria, Australia; University of Texas at Dallas, UNITED STATES

## Abstract

**Background:**

Many common disorders across the lifespan feature impaired working memory (WM). Reported benefits of a WM training program include improving inattention in daily life, but this has not been evaluated in a meta-analysis. This study aimed to evaluate whether one WM training method has benefits for inattention in daily life by conducting a systematic review and meta-analysis.

**Methods:**

We searched Medline and PsycINFO, relevant journals and contacted authors for studies with an intervention and control group reporting post-training estimates of inattention in daily life. To reduce the influence of different WM training methods on the findings, the review was restricted to trials evaluating the Cogmed method. A meta-analysis calculated the pooled standardised difference in means (SMD) between intervention and control groups.

**Results:**

A total of 622 studies were identified and 12 studies with 13 group comparisons met inclusion criteria. The meta-analysis showed a significant training effect on inattention in daily life, SMD=-0.47, 95% CI -0.65, -0.29, p<.00001. Subgroup analyses showed this significant effect was observed in groups of children and adults as well as users with and without ADHD, and in studies using control groups that were active and non-adaptive, wait-list and passive as well as studies using specific or general measures. Seven of the studies reported follow-up assessment and a meta-analysis showed persisting training benefits for inattention in daily life, SMD=-0.33, 95% CI -0.57 -0.09, p=.006. Additional meta-analyses confirmed improvements after training on visuospatial WM, SMD=0.66, 95% CI 0.43, 0.89, p<.00001, and verbal WM tasks, SMD=0.40, 95% CI 0.18, 0.62, p=.0004.

**Conclusions:**

Benefits of a WM training program generalise to improvements in everyday functioning. Initial evidence shows that the Cogmed method has significant benefits for inattention in daily life with a clinically relevant effect size.

## Introduction

Many common disorders across the lifespan feature impairments in working memory (WM) performance, including premature birth [1], ADHD [2,3], cancer treated with radiation [4], acquired head injuries such as stroke [5] and schizophrenia [6]. These impairments are concerning because WM, the ability to temporarily hold information in mind and work with it, is a core cognitive function. It is consistently shown to be important for aspects of everyday functioning including maintaining attention in daily activities [7] and academic achievement, in particular mathematical performance [8,9].

One way to improve WM is through targeted computerised training. A number of WM training methods have been developed (e.g., Cogmed [10] CogniFit [11], Jungle Memory [12], N-back training [13], complex span training [14,15]), and a rapidly increasing number of randomised controlled trials are evaluating the benefits of the different methods. Together the research shows that a WM training program can improve performance on non-trained WM tasks, described as *near transfer effects* (for systematic reviews see: [14,16] for narrative reviews see: [17–19]). The conclusion that WM performance can be increased through training was challenged based on theoretical concerns regarding the concept of WM performance [18,20]. The researchers propose that commonly used span tasks (such as digit and spatial span tasks) employed to evaluate a WM training program are inadequate estimates of WM. However, transfer effects have been subsequently observed following training on complex span tasks that have been proposed by the researchers to better estimate WM, further demonstrating that a WM training program can improve performance on non-trained complex WM tasks (for a review see [21]; see also [15]).

In a previous meta-analysis of WM training methods, Melby-Lervåg and Hulme [16] showed a significant and large training effect for verbal WM and a significant and moderate training effect for visuospatial WM. Subgroup analyses showed that the observed benefits varied across the training programs. For visuospatial WM, the benefit of WM training methods was statistically significant, with the effectiveness of the Cogmed program two to three times higher than the effectiveness of other methods included in the analysis (Jungle Memory, CogniFit, Other). This finding highlights that “working memory training” is not a unitary concept.

Given the importance of WM for everyday functioning, including academic achievement [22–25] and maintaining attention in daily activities [7], there has been increasing interest and debate about the generalising benefits of a WM training program [17,20]. Initial reports provide some suggestion that benefits of a WM training program can generalise, with some studies reporting *far transfer effects*, including: i) improved performance in the lab or clinic on tasks that require WM, such as reasoning [13] and reading comprehension [26], and ii) improved functioning in daily life, such as reducing symptoms associated with a disorder, such as inattention in daily activities [10,27–35]. Establishing generalising benefits for everyday functioning would have exciting implications with both theoretical and clinical significance, especially for ADHD where impaired WM and inattentive behaviour are considered core features of the disorder [3]. So far, no meta-analysis has evaluated the benefits of a WM training program for inattention in daily life.

The primary aim of this study was to evaluate whether a WM training program improves inattention in daily life by conducting a systematic review and meta-analysis of the literature. To reduce the influence of the different WM training programs on the findings, we restricted the review to trials evaluating the Cogmed method, where training is performed as described by Klingberg et al. [10]. The Cogmed program involves computerised adaptive training of WM in 20 or more sessions over a 5-week period. Each session involves training on verbal and visuospatial WM tasks. There are three age-specific versions of the program: JM for preschoolers (4 to 7 years)—requires training for 10 to 15 minutes each session, RM and QM for children, adolescents and adults (7 years and older)—require training for 30 to 45 minutes each session. The three versions are similar, with only the user-interface differing across the versions and the JM version does not include the verbal training tasks that are included in the RM and QM versions. Reinforcement is built into the program, such as small weekly rewards for completing the training sessions. A training aide (typically a teacher or parent) supervises the user to ensure task adherence and breaks are taken. A certified training coach monitors the training by tracking the user’s progress online. The coach has weekly meetings with the user and training aide to review training progress, solve any difficulties with task adherence and ensure compliance, which is an important part of the overall effectiveness of cognitive training.

## Materials and Methods

The PRISMA (Preferred Reporting Items for Systematic Reviews and Meta-Analyses) guidelines (www.prisma-statement.org) were used to identify studies to include in the meta-analysis (See S1 PRISMA Checklist).

### Study search

The studies were identified by searching the electronic databases Medline (Ovid) and PsycINFO (ProQuest) using broad keyword searches: “*working memory training*” or “*working memory* and *training*”. The searches were restricted to English language articles of humans published from February 2005 (when the Cogmed method was first published) to December 2013. Studies were also identified by searching relevant journals for early view publications and we contacted three researchers in the field conducting trials to request unpublished data. Endnote was used to manage the references.

### Study selection

The retrieved articles were reviewed using the following inclusion criteria: (1) The study used the Cogmed WM training method. We only included studies that used the full program (JM, RM or QM) and training schedule (minimum 20 sessions). We did not include studies that used a modified version of the program, e.g. Gibson et al. [36] examined the benefits of Cogmed verbal and visuospatial WM training tasks separately, Bergman-Nutley et al. [37] and Söderqvist et al. [38] examined the benefits of selected Cogmed WM training tasks in combination with non-verbal reasoning training. (2) The study included an intervention and control group using a randomised or non-randomised design. We only included studies that used a passive control group (the group continued with treatment as usual/ no new treatment provided), waitlist or active and non-adaptive control group, where the aim of the study was to evaluate the effectiveness of Cogmed compared with no treatment. We did not include studies that used an active and adaptive control group, where the group received treatment that was designed to improve functioning in a domain closely associated with working memory. We excluded one study with an active and adaptive control group: Gray et al. [39] examined benefits of the Cogmed method compared with benefits of an adaptive math-training program, and WM and math are highly correlated [23–25]. Although it is important to compare the effectiveness of treatments, it was not the focus of the current meta-analysis. (3) The study reported estimates of inattention in daily life pre- and post-training for intervention and control groups. We did not include studies where time between training completion and the follow-up assessment differed for intervention and control groups, e.g. Ludqvist et al. [40] collected estimates of inattention in daily life for the intervention group 20 weeks after training completion and for the control group 4 weeks after training. (4) The study reported means, standard deviations and sample sizes for estimates of inattention in daily life, so that effect sizes could be calculated, and in the case that this information was not reported we contacted authors to obtain information. We contacted the authors of three studies to obtain additional information about post-training scores: one study was not included in the primary analysis [41] because the authors indicated that there was too much missing data to report scores, and one study was not included in the long-term follow up analysis [30] because at the time of submission the information required to perform the analysis had not been obtained. (5) The study data had not been previously reported, to avoid duplication. (6) We considered studies of participants of any age (children, adolescents, adults) and status (healthy, clinical diagnosis, impaired WM). We did not include studies that recruited participants differing on these characteristics, e.g. Løhaugen et al. [35] compared a group of adolescents born extremely low birth weight who had completed the Cogmed program with a control group of adolescents born normal birth weight. It was therefore inappropriate to compare groups on post-training estimates of inattention in daily life because groups differed at baseline on important variables.

Initially titles and abstracts were reviewed for inclusion criteria and then full articles. Articles were reviewed by MSS and TK and excluded if they did not meet all criteria. Any discrepancies were resolved by discussion.

### Coding measures of inattention in daily life

The primary outcome of interest was inattention in daily life. The measures used by studies to estimate inattention in daily life were coded independently by MSS and TK, with any discrepancies resolved by discussion. When a study reported more than one estimate we included the best measure, which was determined using the following order of criteria: (i) Specific measure: in the case that several specific measures were reported we chose the measure most commonly reported in other included studies (e.g. ratings on the Inattention subscale of the DSM-IV ADHD criteria, DuPaul ADHD rating scale, Conners, and Disruptive Behavior Disorders Rating Scale). For example, Since 5 of the identified studies reported parent ratings and only 3 of the studies reported teacher ratings, we preferentially included the parent rating as the primary measure of interest for studies of children and adolescents. Only when parent ratings were not available, teachers ratings were included [34].; (ii) General measure: in the case that several general measures were reported then the measure most commonly reported in other studies was included (e.g. summary scores that included ratings of inattentive symptoms such as the ADHD subscale of the DSM IV ADHD criteria or Conners, the Inattention/Overactivity subscale of the Conners, the Attentional Control subscale from the DuPaul ADHD ratings scale, and the total score of the Cognitive Failures Questionnaire). For example, teacher ratings on the Attentional Control scale from the DuPaul ADHD rating scale rather than teacher ratings on the Total score from the Strengths and Difficulties Questionnaire were included in analyses [34]. Our approach to include both specific and general measures of inattention in daily life acknowledges that behaviour is complex and it is difficult to estimate discrete aspects of behaviour.

### Coding measures of working memory performance

Analyses were performed to confirm that benefits of the Cogmed method for inattention in daily life were accompanied by improvements on WM measures that were similar to the training tasks, reflecting near-transfer. The measures were coded independently by MSS and TK as estimates of visuospatial WM or verbal WM, with any discrepancies resolved by discussion. When a study reported more than one estimate of visuospatial WM or verbal WM we included the best measure, which was determined using the following order of criteria: (i) Specific measure: in the case that several specific measures were reported we included the measure most commonly reported in other included studies (e.g. visuospatial WM: Spatial span backwards, CANTAB Spatial WM, Symbolic WM; verbal WM: Digits backwards, AWMA Listening recall, WRAML Verbal WM).; (ii) General measure: in the case that several general measures were reported we included the measure most commonly reported in other included studies (e.g. visuospatial WM: Span board total score; verbal WM: Digit Span total score).

### Data extraction

We developed a data extraction form based on the Cochrane Consumers and Communication Review Group’s data extraction template. MSS extracted the estimates of inattention in daily life and WM performance, methodological and participant characteristics of the included studies and an independent person checked these extracted data.

### Statistical analyses

Analyses were performed using Review Manager 5.2 [42]. The primary outcome measure of effect size was the pooled standardised difference in means (SMD, which allows comparison of different measurement scales) for inattention in daily life between the intervention and control groups after WM training. Review Manager uses Hedge’s adjusted *g* to calculate SMD, which includes an adjustment for small sample bias. Post-training scores were analysed in line with the Cochrane handbook [43]. A random-effects model was used to calculate the pooled as well as individual study effect sizes and their 95% confidence intervals. In some cases scores included in analyses were reversed so that a negative effect always indicated decreased inattention in daily life. An effect size of-0.2 was considered small, -0.5 moderate, and -0.8 large [44]. Heterogeneity (variability in training effects) between studies was evaluated using the I^2^ statistic, which represents the percentage of the variability in the effect size explained by heterogeneity between studies rather than sampling error, where 25% is mild heterogeneity, 50% moderate, and 75% high [45]. Funnel plots are typically used to examine risk of publication bias when more than 10 group comparisons are included in a meta-analysis [43], with the estimated effect size (SMD) plotted against a measure of sample size (standard error) for individual studies. In the current meta-analysis this approach is problematic because SMD is calculated using sample size and therefore SMD and the standard error are correlated. Therefore, the funnel plot for the primary analysis of interest should be interpreted cautiously (see S1 Fig.). Subgroup analyses were performed to examine whether the intervention effect varied in relation to methodological characteristics: (1) Type of measure. Measures used to estimate inattention in daily life were coded as general or specific. (2) Type of control group. The experience of the control group was coded as active and non-adaptive training, wait-list or passive. Subgroup analyses were also performed to determine whether the intervention effect varied in relation to participant characteristics: (1) Age. Participants were coded as children and adolescents or adults. (2) Status. Participants in the study were coded as healthy, diagnosed with ADHD or at-risk for WM impairment (i.e. previous research indicates the study sample is at-risk for WM impairment). Subgroups with at least 4 effect sizes were included in analyses.

Analyses were performed to confirm improvements on visuospatial WM and verbal WM measures after training, indicating benefits of the WM training program identified in the primary analysis were accompanied by near-transfer effects. Here a positive effect size indicated improved WM.

## Results

**Fig 1:**
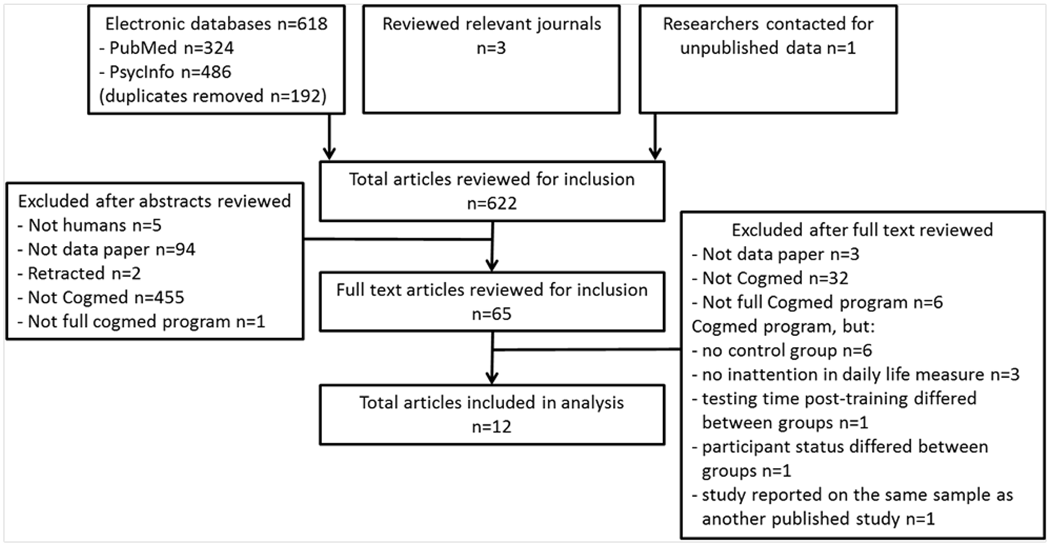
Flow diagram for the systematic search and selection of studies.

A total of 622 articles were identified and reviewed for inclusion: 618 articles were identified by systematically searching electronic databases (192 duplicates), 3 articles were identified by searching relevant journals, and 1 article identified from contacting researchers for unpublished data. The dataset included 12 studies that met inclusion criteria with 13 group comparisons of the Cogmed program. All studies were randomised controlled trials. See Fig. 1 for a flow diagram of study selection and Table 1 for a summary of the methodological and participant characteristics of the identified studies. One study was not included in analyses because inattention in daily life ratings were not reported in the paper due to missing data [46]. The final dataset used in analyses included 11 studies and 12 group comparisons of the Cogmed program.

**Table 1:** Participant and methodological characteristics of identified studies.

Study	Participants			Risk of Bias		Control Group	Training		Testing		Outcome measure		
	Age in years	Status	Recruitment source	Design	Blinded a) Rater b) Tester		Version	Site	Immediate follow-up	Delayed follow-up	Inattentive behaviour (rater)	Visuospatial WM	Verbal WM
Beck 2010* [30]	7–17	ADHD^b^	Private school for students with ADHD and/or LD	RCT	a) baseline, b) NR	Wait-list	RM	Home	1 month	4 months	DSM-IV Inattention (parent)	none	none
Björkdahl 2013+ [41]	22–63	WM impairment (Brain Injury)	Outpatient rehabilitation clinic	RCT	a) NR, b) NR	Passive	QM	NR	1 week	3 months	Working memory questionnaire (self)	none	WAIS-III Digit span backward
Brehmer 2012 [56]	a) 20–30, b) 60–70	Healthy	Newspaper advertisement	RCT	a) yes, b) yes	Active and non-adaptive	QM	Home	NR	3 months	CFQ (self)	WAIS-R Span board backward	WAIS-R Digit span backward
Chacko 2013 [55]	7–11	ADHD	Newspaper advertisements	RCT	a) yes, b) yes	Active and non-adaptive	NR	Home	3 weeks	none	DBD Inattention (parent)	AWMA Spatial recall	AWMA Listening recall
Egeland 2013 [50]	10–12	ADHD^b^	Outpatient clinics at two hospitals	RCT	a) NR, b) yes	Wait-list	NR	School	NR	8 months	DuPaul Inattention (parent)	none	none
Green 2012 [31]	7–14	ADHD^b^ ^,^ ^c^	Advertising, psychologists, psychiatrists, institute tracking	RCT	a) yes, b) yes	Active and non-adaptive	RM	Home	NR	none	Connors ADHD (parent)	none	none
Gropper 2013 [60]	19–52	ADHD^b^ (ADHD/LD)	Student Disability services at 3 post-secondary institutions	RCT	a) NR, b) NR	Wait-list	QM	Home	3 weeks	2 months	CFQ (self)	CANTAB Spatial WM (errors)	WISC-IV Digit span
Grunewaldt 2013 [32]	5–6	WM impairment (VLBW)	Admission records of a hospital NICU	RCT^e^	a) NR, b) yes	Wait-list	JM	Home	1 month	none	DuPaul Inattention (parent)	Spatial span backward^a^	Digit span backward^a^
Hardy 2013 [33]	8–16	WM impairment (Cancer)	Patients of the hospital Division of Pediatric Hematology-Oncology	RCT	a) yes^d^, b) yes	Active and non-adaptive	RM	Home	NR	3 months	Connors Inattention (parent)	WRAML Symbolic WM	WRAML Verbal WM
Klingberg 2005 [10]	7–12	ADHD	Referrals from pediatricians, psychiatrists, special school teachers	RCT	a) yes, b) yes	Active and non-adaptive	RM	Home or school	NR	3 months	DSM-IV Inattention (parent)	WAIS-R Span board	WAIS-R Digit span
Roughen 2011 [34]	15–17	WM impairment (SEB)	Schools	RCT	a) no, b) NR	Passive	RM	School	3 weeks	3 months	DuPaul Attentional Control (teacher)	none	none
Westerberg 2007 [2]	34–65	WM impairment (Stroke)	Stroke Rehabilitation Unit	RCT	a) baseline, b) baseline	Passive	RM	Home	NR	none	CFQ (self)	WAIS-R Span board	WAIS-R Digit span

Note. ADHD, Attention deficit hyperactivity disorder; CFQ, Cognitive Failures Questionnaire; DBD, Disruptive Behaviors Disorders Rating Scale, ELBW, extremely low birth weight; LD, learning disorder; NR, not reported; RCT, randomised controlled trial; SEB, social and emotional behavioural difficulties; VLBW, very low birth weight; WM, working memory

+ not included in analyses because authors did not report inattention ratings due to missing data

* not included in the analysis of delayed effects of training because scores required for the analysis were not reported in the paper and at the time of submission authors had not provided information

^a^ described by authors as a standard neuropsychological test

^b^ some users prescribed stimulant medication

^c^ participants assessed for LD and 1 met criteria

^d^ families informed pre-training there were two versions of the program reflecting different levels of difficulty

^e^ trial is a RCT at post-training testing, and at long-term follow-up is a stepped wedge design

### Inattention in daily life after the training

**Fig 2:**
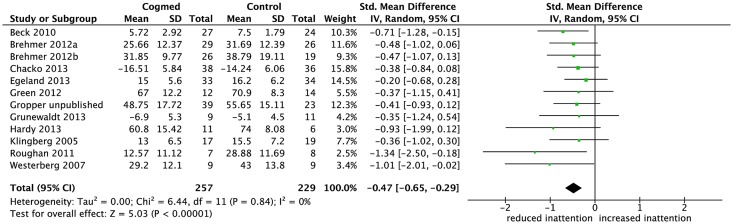
Forest plot for inattention in daily life after the training. The overall pooled effect size (standardised mean difference, displayed as a diamond) as well as individual study effect sizes (displayed as rectangles) and their 95% confidence intervals (represented by horizontal lines) are shown.

Fig. 2 shows the 11 studies with 12 group comparison effect sizes and the pooled effect size comparing estimates of inattention in daily life for the intervention and control groups after completing the WM training program (up to 4 weeks post-training). The effect sizes ranged from-0.20 to -1.34. The meta-analysis showed that the pooled effect size was moderate and significant (SMD = -0.47, 95% CI-0.65, -0.29, p<.00001), with heterogeneity between studies low (I^2^ 0%) and non significant (χ^2^ = 6.44, df 11, p = .84). See supplementary material for funnel plot.

#### Examination of methodological characteristics

Table 2 shows a summary of the subanalyses performed to examine whether the intervention effect varied in relation to methodological characteristics. There was some suggestion of variability in the subgroup effect sizes when studies were grouped based on the type of control group used, but this difference was non significant (p = .84). For studies using an active and non-adaptive control group or a waitlist control group the subgroup effect size was small to moderate and significant. Two studies used a passive control group and therefore this subgroup was not included in the analysis. Studies using a specific measure as well as studies using a general measure to estimate inattention in daily life showed subgroup effect sizes that were moderate and significant, with no significant difference between subgroups.

**Table 2:** Subgroup analyses for the effects of methodological and participant characteristics on inattention in daily life after training.

Subgroups	No. of effect sizes	n	SMD (95% CI)	p	Heterogeneity (I^2^)	Test for subgroup difference
Control group						
Active and non-adaptive	6	253	-0.44 (-0.70, -0.19)	.0005	0%	
Wait-list	4	200	-0.41 (-0.69, -0.12)	.005	0%	χ^2^ = 3.38, p = .84, I^2^ 0%
Measure						
Specific	6	265	-0.42 (-0.66, -0.17)	.0008	0%	
General	6	221	-0.53 (-0.81, -0.26)	.00001	0%	χ^2^ = 0.36, p = .55, I^2^ 0%
Age						
Children and adolescents	8	306	-0.45 (-0.68, -0.22)	.0001	0%	
Adults	4	180	-0.50 (-0.80, -0.20)	.001	0%	χ^2^ = 0.07, p = .79, I^2^ 0%
Status						
ADHD	6	316	-0.39 (-0.62, -0.17)	.0006	0%	
WM impairment	4	70	-0.84 (-1.35, -0.34)	.001	0%	χ^2^ = 2.56, p = .11, I^2^ 60.8%

Note. Results are presented for analyses including subgroups with at least 4 effect sizes, although significance of results did not change when analyses were performed including the passive control subgroup and healthy status subgroup (each with 2 effect sizes); CI, confidence intervals; SMD, stardardised mean difference; WM, working memory

#### Examination of participant characteristics

Table 2 shows a summary of the subgroup analyses performed to examine whether the observed training benefits for inattention in daily life changed for different groups of participants. For studies of children and adolescents as well as studies of adults the subgroup effect sizes were small to moderate and significant, with no significant difference between subgroups. There was variability observed in the subgroup effect sizes when studies were grouped based on participant status, although this difference was not statistically significant (p = .11). For studies of participants diagnosed with ADHD the subgroup effect size was small to moderate and significant. One study with two group comparisons used healthy participants and therefore this subgroup was not included in the analysis. For studies of participants at risk for WM impairment the subgroup effect size was large and significant.

In summary, the meta-analysis indicated a moderate and significant effect of the WM training program on inattention in daily life. This training effect did not change significantly depending on methodological characteristics of the studies we examined (type of measure, type of control group). Furthermore, subgroup analyses showed that the training effect was observed for different groups of participants (children and adolescents as well as adults, participants diagnosed with ADHD or at risk for impaired WM).

### Inattention in daily life following a delay after training

**Fig 3:**
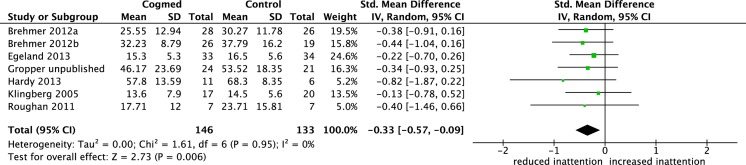
Forest plot for inattention in daily life following a delay after the training. The overall pooled effect size (standardised mean difference, displayed as a diamond) as well as individual study effect sizes (displayed as rectangles) and their 95% confidence intervals (represented by horizontal lines) are shown.

Seven studies included in the primary analysis of interest reported inattention in daily life following a delay after the WM training program (ranging from 2 to 4 months). One study was not included in this analysis because the M and SD were not obtained. Fig. 3 shows the 7 studies with 8 group comparison effect sizes and the pooled effect size comparing estimates of inattention in daily life for the intervention and control groups. The effect sizes ranged from -0.13 to -0.82. The meta-analysis showed that the pooled effect size was small to moderate and significant (SMD = -0.33, 95% CI-0.57, -0.09, p = .006), with heterogeneity between studies low (I^2^ 0%) and non significant (χ^2^ = 1.16, df 6, p = .95).

This analysis provides initial evidence for persisting benefits of the WM training program for inattention in daily life. Our results suggest that ongoing benefits might be slightly reduced, however it should be noted that the analysis was based on 8 studies included in the primary analysis that conducted follow-up assessments.

### Working memory performance after the training for included studies

**Fig 4:**
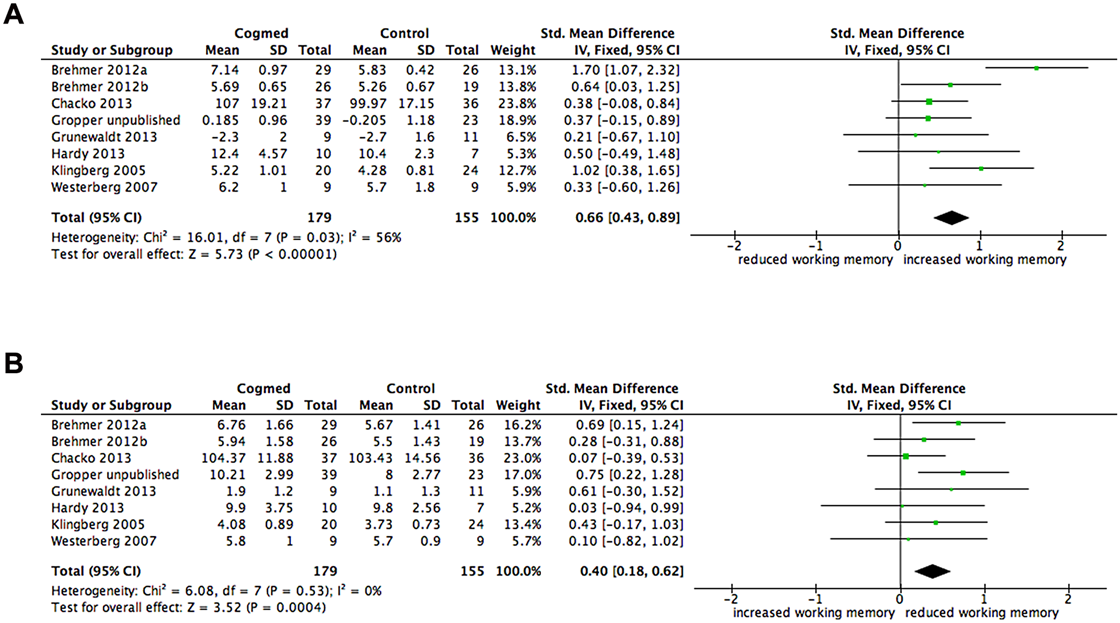
Forest plot for (a) visuospatial working memory performance and (b) verbal working memory performance after the training. The overall pooled effect size (standardised mean difference, displayed as a diamond) as well as individual study effect sizes (displayed as rectangles) and their 95% confidence intervals (represented by horizontal lines) are shown.

Seven studies (with 8 group comparisons) included in the primary analysis of interest reported visuospatial WM performance for the intervention and control groups after the WM training program. Fig. 4 shows the pooled effect size comparing estimates of visuospatial WM performance for intervention and control groups after completing training was large and significant (SMD = 0.67, 95% CI 0.31, 1.02, p = .0002), with heterogeneity between studies moderate (I^2^ 56%) and significant (χ^2^ = 16.01, df 7, p = .03).

The same seven studies (with 8 group comparisons) also reported verbal WM performance for the intervention and control groups after the WM training program. Fig. 4 shows the pooled effect size comparing estimates of verbal WM performance for intervention and control groups after completing training was moderate and significant (SMD = 0.40, 95% CI 0.18, 0.62, p = .0004), with heterogeneity between studies low (I^2^ 0%) and non significant (χ^2^ = 6.08, df 7, p = .53).

In summary, the meta-analysis indicated that generalising benefits of the WM training program for inattention in daily life were observed in the context of a significant effect of the WM training program on both near-transfer measures of visuospatial WM and verbal WM performance.

All results were similar when analyses were performed using a fixed-effects model and results obtained that were significant using the random-effects model remained significant using the fixed-effects model.

## Discussion

The major finding of this meta-analysis is that benefits of a WM training program generalise to improvements in everyday functioning. We showed that inattention in daily life was significantly improved after completing the Cogmed program compared with a control program. The training effect was-0.47 SMD. Whether a significant and moderate training effect on inattention in daily life is adequate given the time and effort of a training program is open to discussion. Many existing pharmacological treatments for behavioural aspects of common clinical disorders, such as antidepressant medications [47] and cholinesterase inhibitors for Alzheimers disease [48], have an effect size of around 0.3 or less. In education, interventions with an effect size of 0.25 are considered useful [49]. Of particular clinical importance, initial evidence here shows that the benefit of Cogmed for inattention in daily life was observed for a range of participant groups. Children and adolescents as well as adults benefitted from the training, highlighting plasticity across a wide age range. Individuals diagnosed with ADHD as well as individuals at risk of WM impairments showed reduced inattention in daily life after the training. Furthermore, initial evidence here shows that the training effect was observed regardless of methodological characteristics examined, including whether a commonly used and validated general or specific measure was used to estimate inattention in daily life and whether the control group in the study design was active and non-adaptive or wait-list. We also showed that benefits of the training for inattention in daily life persisted after the training and remained significant at 2 to 8 months after training, with a small to moderate effect size (SMD = -0.33). Importantly, the generalising (far-transfer) effect of the Cogmed method to inattention in daily life was observed in the context of expected moderate to large and significant improvements in visuopatial WM and verbal WM performance (near-transfer effects). The moderate and significant training effect on inattentive behaviour found in this meta-analysis is not inconsistent with findings of non-significant effects reported in some of the small sample studies included in the meta-analysis (e.g. Egeland et al. [50]). The low statistical power in most of the training studies published to date highlights the need for large sample studies, meta-analyses in the future, and to acknowledge and estimate the risk of type-II errors when reporting small sample trials.

Our finding of improved inattention in daily life after completing a WM training program is consistent with the documented association between WM impairments and inattentive behaviour. This association has been examined in non-clinical samples of children and adolescents [7] as well as clinical groups including children diagnosed with ADHD [51], the most common childhood disorder [52]. Our meta-analysis provides initial evidence that trajectories of inattentive behaviour can be improved through a WM training program. This could have related benefits for the child and family, as well as teachers. However, a limitation of this study is the small number of studies included in the analysis to examine persisting and long-term benefits of training. In the current meta-analysis eight of the included studies conducted follow-up, varying from 2 to 8 months post-training. The next critical step will be to adequately establish long-term benefits for inattention in daily life, which will become evident with the publication of the increasing number of trials conducting long-term follow-up.

A main strength of this meta-analysis is the focus on one specific WM training method. Considerable variability in the near-transfer benefits of various WM training programs was highlighted recently in a meta-analysis of WM training [16]. The difference in effect sizes for the various methods was found to be statistically significant for visuospatial WM, the aspect of WM characteristically impaired in ADHD [53]. Thus, evaluating the benefits of a specific WM training program reduces the amount of introduced variance in a meta-analysis because WM training is not a unitary concept. In our analysis we examined potential sources of variability discussed in the training literature, including participant characteristics (age, status) and methodological characteristics related to study quality (type of measure, type of control group). We acknowledge that subanalyses were based on small numbers of trials and we suggest that results at this stage provide initial insight.

Participant characteristics of the included studies are important to consider when interpreting findings of this meta-analysis. A limitation of the current study is the wide range in age groups that were formed to examine the effects of participant age (children and adolescents group ranged from 5 to 17 years, adults group ranged from 20 to 70 years). This largely reflects the wide age ranges of participants in the included studies, which might have limited detection of specific age effects. User motivation is a potential source of variability across studies [53] that was not examined, but this was not reported in any of the included studies and so far has been rarely assessed in trials evaluating a WM training method. One study that directly measured motivation did not find a significant association with improvement on a WM training program [54]. Training effects might vary across users diagnosed with ADHD, depending on severity (e.g. prescribed medication) and co-morbidity (especially oppositional defiant disorder, see [55]). Additional trials will be required to examine the influence of these individual characteristics.

Methodological characteristics of the included studies in this meta-analysis should also be considered when interpreting the findings. It is important to note the logic of experimental research is that a control group should control for as many influencing factors as possible, in order to isolate the factor leading to differences in outcomes between the treatment and control condition. A control group should thus control for the possible effects of factors such as test-retest, passage of time, expectancy, and motivation. Even when a control condition is created with this in mind, possible confounds in a control condition should be considered. In studies evaluating a WM training program compared with a non-adaptive training program as a control condition there have been no indications of confounds such as increased drop-out [10,56] or differences in motivation when it is directly estimated [37]. A study by Thorell et al. [57] evaluating a WM training program compared with a control group using computer games obtained results that were qualitatively similar to studies using a non-adaptive control group, with the WM training group improving significantly on tasks measuring WM and attention. In the same study, improvements were observed for a group training on inhibitory tasks, highlighting that the training effect was specific to the WM tasks. Together these studies indicate that a non-adaptive control group is an adequate control condition.

It could be considered a limitation that we did not include in this meta-analysis a study employing a control group that received a treatment designed to improve functioning in a domain closely associated with WM. Gray et al. [39] compared benefits of Cogmed RM with adaptive math training in adolescents with specific learning difficulties and coexisting ADHD. Participants were attending a school for adolescents with a history of not responding to prior interventions, including pharmacological treatment and special education, and might therefore be less response to cognitive training. Using the same procedure for selecting outcome measures and calculating effect sizes in this meta-analysis, Cogmed compared with math training showed no significant differences for behaviour ratings (a small, non-significant effect on inattention in daily life; IOWA Conners Parent Rating Scale, Inattention/Overactivity subscale: SMD = 0.27, 95% CI-0.29, 0.84), a moderate and significant positive effect on verbal WM (WISC-IV Digit Span Backwards: SMD = 0.52, 95% CI-0.04, 1.09) and a moderate to large significant positive effect on visuospatial WM (CANTAB Spatial WM: SMD = 0.60, 95% CI 0.02, 1.17). However, moving forward, it will be important for trials, such as that of Gray et al., to compare the effectiveness of Cogmed with other cognitive training programs in order to establish a gold standard method to enhance WM and associated functions. It is important to acknowledge that for the studies included in this meta-analysis, independent researchers chose the control group that was their best option and the studies were published in peer-reviewed journals. Potential methodological sources of variability not examined in the current meta-analysis include how participants were recruited (representative cohort or convenience sample) and where the training was performed (home or school setting).

Relevant to the discussion here of potential sources of variability in the training effect is the use of ratings scales to estimate inattention in daily life. It is important to note that rating scales is a limitation of trials that are not blinded. In the current review around half of the identified trials were reported by authors to be blinded but this information was not provided for many of the included trials (see Table 1). We used ratings provided by different informants using commonly employed and validated measures to estimate inattention in daily life. In this meta-analysis we preferentially included parent ratings for studies of children and adolescents because this was the most commonly reported measure across the identified studies. Included in the meta-analysis were parent ratings for 7 studies and teacher ratings for 1 study of children and adolescents, and self-ratings for 4 adult studies (see Table 1). The inclusion of estimates from both parent and teacher ratings could be considered a limitation of the meta-analysis. It is well established that parent and teacher ratings of inattention in daily life correlate modestly [58,59]. This pattern of association of parent and teacher ratings has been observed in randomised controlled trials evaluating changes in inattention in daily life after completing a WM training program [10,30,39], but there is currently no consensus in the literature on what the discrepancy reflects. In the current review, we performed an analysis of interest focusing on teacher ratings reported in the included studies. Only 4 of the included studies included teacher ratings of inattention and consistent with the positive effects of training on inattention observed in our primary analysis of interest, results provide an initial suggestion of training benefits for inattention in daily life rated by teachers (SMD = -0.30, 95% CI-0.61, 0.01, p = 0.05, I^2^ 11%, (χ^2^ = 3.36, df 3, p = 0.34).

Gray et al. [39] in their randomised controlled trial of the Cogmed program attempted to understand the observed discrepancies in parent and teacher ratings on the IOWA Conners for adolescents with ADHD/LD who completed training at school. Parents reported significant improvements in behaviour, but not teachers. WM Index improvement (a summary of change in performance on Cogmed training tasks) significantly correlated with parent ratings but not teacher ratings of change in inattention in daily life. This finding provides some suggestion that parent ratings are sensitive in detecting change in inattention in daily life. It will be important for future research to examine this further and establish how to interpret discrepancies in parent and teacher ratings of change. Green et al. [31] speculate that teacher ratings might not be sensitive in detecting positive changes in on-task behaviour, with their attention dispersed across a group of students and often selectively pulled towards behavioural management. In an attempt to address this potential limitation of teacher ratings, Green et al. estimate behavioural change using the Restricted Academic Situation Task (RAST). This laboratory measure requires the child to play with a toy and then complete a basic written academic task with the toy in the room. The frequency and type of off-task behaviour during the academic task, which might not be salient to a teacher, is quantified by observers blinded to treatment allocation. Using these objective measures, Green et al. observed a significant reduction in overall inattentive behavior, with strongest effects on rather subtle behaviors such as "looking away" during the academic task in the training group compared with the control group. Keeping focused on the task at hand is a highly relevant aspect of attentive behaviour that a teacher might have difficulty observing for all children in a classroom. Virtual reality tasks, such as the virtual classroom, which provide an objective estimate of inattention in daily life, could offer complementary insight into changes in everyday functioning after completing a WM training program.

## Conclusions

In conclusion, this meta-analysis shows that benefits of a WM training program generalise to improvements in everyday functioning. Specifically, the Cogmed method has a clinically relevant benefit for inattention in daily life after training based on commonly used and validated measures. Initial evidence shows that this robust benefit was observed for groups of children and adults as well as individuals diagnosed with ADHD and impaired WM. It will be important for future trials to evaluate long-term benefits in order to reliably determine the persisting benefits of a WM training program.

## Supporting Information

S1 PRISMA Checklist PRISMA Checklist. (DOC)

S1 Fig Funnel plot for inattention in daily life after training. The overall pooled effect size (standardised mean difference, represented by the vertical line) and its 95% confidence intervals (represented by diagonal lines) are presented, showing the expected distribution of studies in the absence of heterogeneity or of selection biases. The funnel plot has been described as a means of displaying small study effects (Cochrane Collaboration 2011). In S1 PRISMA Checklist, the three trials with smallest samples show the largest effect sizes and are scattered towards the bottom left of the plot: Hardy 2013, Roughan 2011, Westerberg 2007. Asymmetry in a funnel plot might reflect: 1) Selection biases, 2) True heterogeneity, 3) Data irregularities, 4) Artifact, and 5) Chance (The Cochrane Collaboration, 2011). Two of these studies were included in analyses of other outcomes examined in the current study, visuospatial working memory and verbal working memory, and did not show the largest effect sizes (see Fig. 4). In an analysis of interest, when the three trials were excluded the training effect remained significant and the effect size moderate.
(TIF)
