# Correction: Benefits of a Working Memory Training Program for Inattention in Daily Life: A Systematic Review and Meta-Analysis

**DOI:** 10.1371/journal.pone.0167373

**Published:** 2016-11-22

**Authors:** Megan Spencer-Smith, Torkel Klingberg

In the original article, two values from the studies by Chacko et al (2013) and Grunewaldt et al (2013) were incorrectly coded in the main analysis examining the effect of Cogmed on inattention in daily life. With the correct coding the overall effect size of the intervention on inattention in daily life compared with a control group is SMD = -0.37 and the 95% confidence intervals are -0.63 to -0.11. As previously reported, this overall effect is significant (p =.005). There were no significant differences between any of the subgroups, as previously reported. Details of these corrected analyses are provided below, together with further consideration of publication bias and the small sample sizes of the included studies.

For visuospatial working memory, the Grunewaldt et al (2013) study was incorrectly coded and with the correct coding the overall effect size of the intervention on visuospatial working memory compared with a control group is SMD = 0.62, the 95% confidence intervals are 0.24 to 1.01, and this overall effect is significant (p =.001). For verbal working memory, the Gropper et al (unpublished) study was incorrectly coded and with the correct coding the overall effect size of the intervention on verbal working memory compared with a control group is SMD = 0.41, the 95% confidence intervals are 0.19 to 0.63, and this overall effect is significant (p =.0003).

## Inattention in daily life after the training

The main analysis of interest showed that the pooled effect size comparing estimates of inattention in daily life outcome for the intervention and control groups was small to medium and significant (SMD = -0.37, 95% CI -0.63 to -0.11, p =.005), with heterogeneity between studies moderate (I^2^ 45%) and not significant (p =.05). See Fig 3 below. Of interest, Chi Square test for heterogeneity (I^2^) is considered to have low power and therefore non significance is not assumed to reflect homogeneity, and a higher significance level (e.g. p <.10) is sometimes considered for determining significance (Cochrane Handbook).

**Fig 3:**
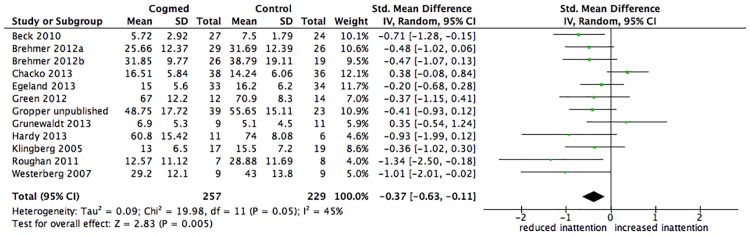
Forest plot for inattention in daily life after training. The overall pooled effect size (standardised mean difference, displayed as a diamond) as well as individual study effect sizes (displayed as rectangles) and their 95% confidence intervals (represented by horizontal lines) are shown.

Subgroup analyses were performed to provide initial insight into whether the intervention effect observed in the main analysis differed across situations, including methodological characteristics of the studies and participant characteristics. There were no significant differences between subgroups and there was a tendency for a moderate degree of heterogeneity across studies, see Table 2 below. Subgroup analyses were based on between study comparisons, not randomised comparisons, and for this reason the Cochrane Group and others suggest care when interpreting results. Guidelines for interpreting subgroup analyses suggest magnitude of effects to be compared rather than the statistical significance of the results within separate subgroups.

**Table 2:** Subgroup analyses for the effects of methodological and participant characteristics on inattention in daily life after training.

Subgroups	No. of effect sizes	SMD (95% CI)	p	Heterogeneity I^2^	Test for subgroup difference
Control group					
Active and non-adaptive	6	-0.29 (-0.66, 0.08)	.13	51%	
Wait-list	4	-0.32 (-0.67, 0.03)	.07	32%	χ^2^ = 0.01, p =.91, I^2^ 0%
Measure					
Specific	6	-0.20 (-0.61, 0.21)	.33	60%	
General	6	-0.53 (-0.81, -0.26)	.0001	0%	χ^2^ = 1.70, p =.19, I^2^ 41.2%
Age					
Children and adolescents	8	-0.31 (-0.68, 0.07)	.11	58%	
Adults	4	-0.50 (-0.80, -0.20)	.001	0%	χ^2^ = 0.63, p =.43, I^2^ 0%
Status					
ADHD	6	-0.25 (-0.58, 0.08)	.14	51%	
Working memory impairment	4	-0.69 (-1.47, 0.10)	.09	57%	χ^2^ = 1.03, p =.31, I^2^ 2.6%

Note. Results are presented for analyses including subgroups with at least 4 effect sizes; CI, confidence intervals; SMD, standardised mean difference

## Risk of publication bias

The authors attempted to reduce publication bias by searching comprehensively for studies that met eligibility criteria and contacted researchers in the field for unpublished data. A limitation of this review is that the authors did not search trial registries for trials that have not been published. Of the 11 included studies, 3 were reported as registered clinical trials (Chacko et al., 2013; Egeland et al., 2013; Grunewaldt et al., 2013).

There is currently no consensus on a method for detecting and addressing publication bias. In the original article, the authors presented a funnel plot for the main analysis, a method often used to examine publication bias when more than 10 studies are included. The authors acknowledged the limitations of using funnel plots more generally, such as the assumption that asymmetry indicates publication bias, and more specifically in the case of the authors’ analysis given the authors report SMD as the estimated effect size. A commonly used method to detect and quantify the amount of bias captured by the funnel plot asymmetry is Egger’s regression test, which is better described as detecting small study effects (Cochrane Handbook). Results of this test performed in Stata 14 provide weak evidence for the presence of small study effects (bias coefficient = -2.41, 95% CI -5.18 to 0.37, p =.082). Therefore, the authors have not performed an analysis to address publication bias, such as the trim-and-fill method.

## Small sample sizes of included studies

The authors’ review identified trials with relatively small sample sizes for inclusion in the meta-analysis. This limitation of small sample size, which raises concerns about the power to detect a statistically significant effect, largely reflects the current cognitive training literature. Although an advantage of performing a meta-analysis is to increase the statistical power of the analysis by pooling the samples, the inclusion of only small sample studies is a limitation that the authors acknowledge. Outcomes for the intervention and control groups were compared using post-treatment ratings of inattention and the small sample sizes of the included trials can have an influence in the results if there are imbalances in baseline scores, even when they are randomised controlled trials. References to benefits or improvements in the original article should therefore be referred to as “effects”. Publication bias is a risk when small sample size studies are included in a meta-analysis, and in this context standard methods of detecting possible publication bias are not likely to detect even substantial publication bias. A meta-analysis is not a substitute for large studies, and it is necessary for future registered trials to recruit large samples. Given the small samples of the identified trials in the current meta-analysis, one approach might have been to synthesise the data qualitatively. Results of the authors’ meta-analysis should be interpreted in the context of its limitations, as with all meta-analyses.

## Working memory after the training

Fig 4 below presents results summarising the effect of Cogmed on visuospatial and verbal working memory performance compared with a control group. The pooled effect size comparing visuospatial working memory performance for the intervention and control groups was medium to large (SMD = 0.62, 95% CI 0.24 to 1.01, p =.001), with heterogeneity between studies moderate to high (I^2^ 63%) and non-significant (p =.009). The pooled effect size comparing verbal working memory performance for the intervention and control groups was small to medium (SMD = 0.41, 95% CI 0.19 to 0.63, p =.0003) and there was little evidence of heterogeneity between studies (I^2^ 0%, p =.45).

**Fig 4:**
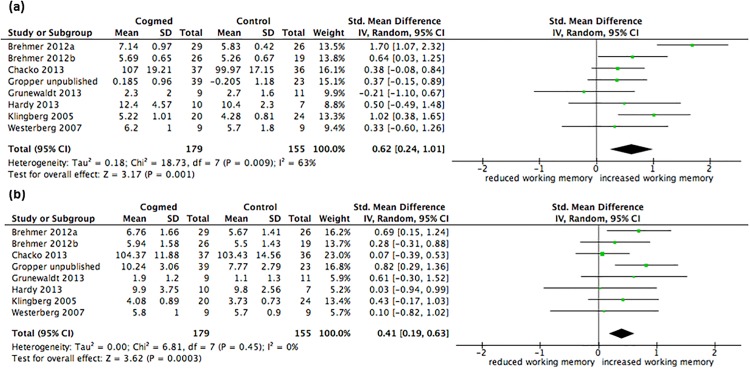
Forest plots for (a) visuospatial working memory performance and (b) verbal working memory performance after training. The overall pooled effect size (standardised mean difference, displayed as a diamond) as well as individual study effect sizes (displayed as rectangles) and their 95% confidence intervals (represented by horizontal lines) are shown.
