# Supplementary figures and images for: Benefits of a Working Memory Training Program for Inattention in Daily Life: A Systematic Review and Meta-Analysis
Source: PLoS One. 2015 Mar 20;10(3):e0119522. doi: 10.1371/journal.pone.0119522 (PMC4368783; doi:10.1371/journal.pone.0119522)

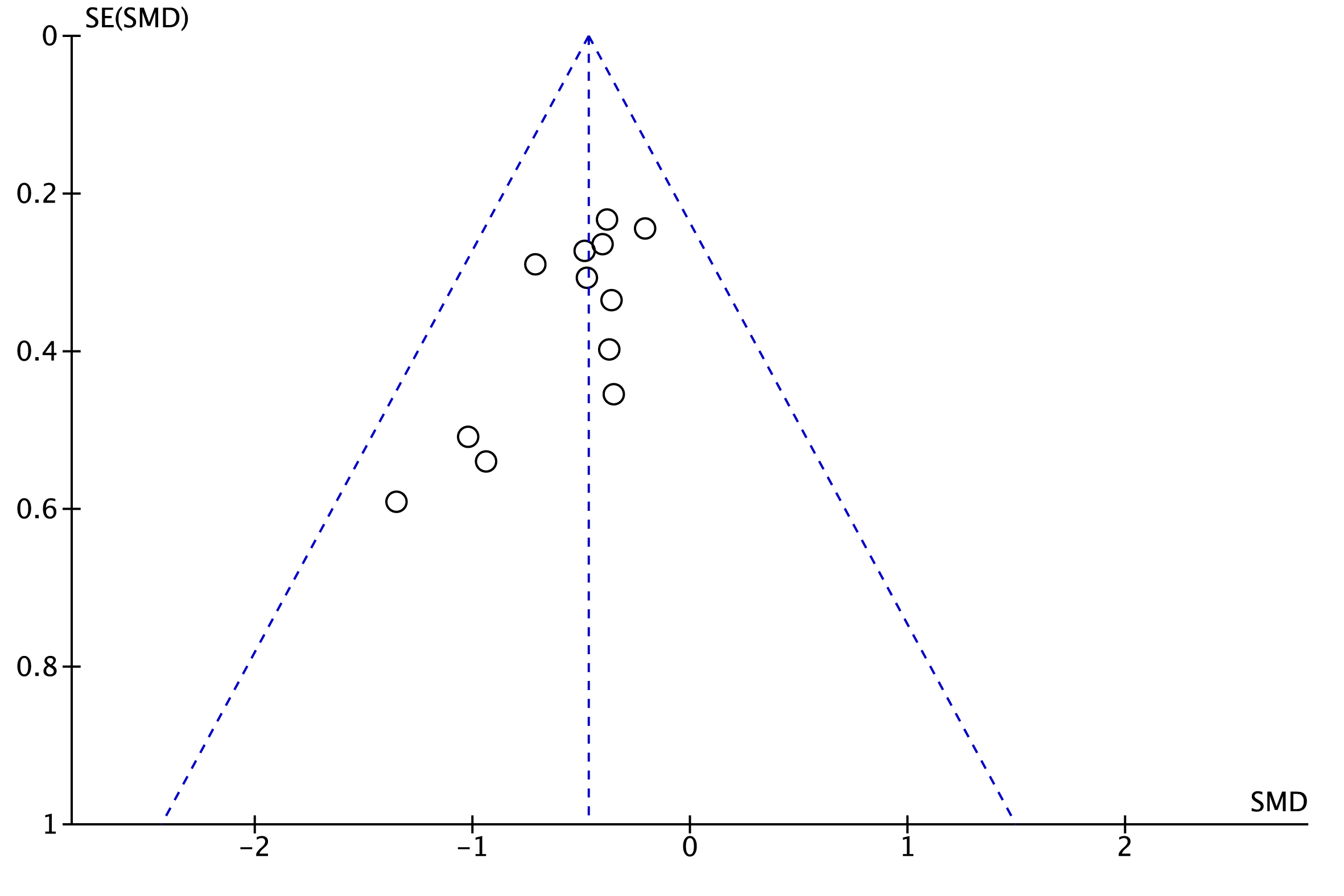

Supplement: S1 Fig — The overall pooled effect size (standardised mean difference, represented by the vertical line) and its 95% confidence intervals (represented by diagonal lines) are presented, showing the expected distribution of studies in the absence of heterogeneity or of selection biases. The funnel plot has been described as a means of displaying small study effects (Cochrane Collaboration 2011). In S1 PRISMA Checklist, the three trials with smallest samples show the largest effect sizes and are scattered towards the bottom left of the plot: Hardy 2013, Roughan 2011, Westerberg 2007. Asymmetry in a funnel plot might reflect: 1) Selection biases, 2) True heterogeneity, 3) Data irregularities, 4) Artifact, and 5) Chance (The Cochrane Collaboration, 2011). Two of these studies were included in analyses of other outcomes examined in the current study, visuospatial working memory and verbal working memory, and did not show the largest effect sizes (see Fig. 4). In an analysis of interest, when the three trials were excluded the training effect remained significant and the effect size moderate. (TIF) [file pone.0119522.s002.tif]
